# Antihypertensive and Lipid-Lowering Medication Adherence in Young Adults With Youth-Onset Type 2 Diabetes

**DOI:** 10.1001/jamanetworkopen.2023.36964

**Published:** 2023-10-04

**Authors:** Ruth S. Weinstock, Paula M. Trief, Brian K. Burke, Hui Wen, Xun Liu, Seth Kalichman, Barbara J. Anderson, Jane D. Bulger

**Affiliations:** 1 Department of Medicine, State University of New York Upstate Medical University, Syracuse; 2 Department of Psychiatry and Behavioral Sciences, State University of New York Upstate Medical University, Syracuse; 3 Biostatistics Center, George Washington University, Rockville, Maryland; 4 Department of Psychological Sciences, University of Connecticut, Storrs; 5 Department of Pediatrics, Baylor College of Medicine, Houston, Texas

## Abstract

**Importance:**

Youth-onset type 2 diabetes is associated with early development of chronic complications. Treatment of elevated blood pressure (BP), nephropathy, and dyslipidemia are critical to reduce morbidity. Data are needed on adherence to BP- and lipid-lowering medications in young adults with youth-onset diabetes.

**Objective:**

To assess adherence and factors associated with adherence to BP- and lipid-lowering medications in young adults with youth-onset type 2 diabetes and diagnoses of hypertension, nephropathy, or dyslipidemia.

**Design, Setting, and Participants:**

This cohort study measured medication adherence with 3 monthly unannounced pill counts at 2 time points 1 year apart during iCount, conducted during the last years (2017-2019) of the observational phase of the Treatment Options for Type 2 Diabetes in Adolescents and Youth study. Psychosocial factors associated with medication adherence were examined. Participants included individuals with youth-onset type 2 diabetes with hypertension, nephropathy, or dyslipidemia receiving diabetes care in their communities. Data were analyzed from September 2022 to September 2023.

**Main Outcomes and Measures:**

The main outcome was BP- and lipid-lowering medication adherence, with low adherence defined as using less than 80% of pills and high adherence, at least 80% of pills. Psychosocial factors were measured using the Beliefs about Medicines Questionnaire and Material Needs Insecurities Survey.

**Results:**

Of 381 participants in iCount, 243 participants (mean [SD] age, 26.12 [2.51] years; 159 [65.43%] women) with hypertension, nephropathy, or dyslipidemia were included in analysis. Among 196 participants with hypertension or nephropathy, 157 (80.1%) had low adherence. Participants with low adherence, compared with those with high adherence, were younger (mean [SD] age, 25.99 [2.41] vs 27.26 [2.41] years; *P* = .005), had higher glycated hemoglobin A_1c_ (mean [SD], 10.33% [2.66 percentage points] vs 8.85% [2.39 percentage points]; *P* = .001), shorter diabetes duration (mean [SD], 12.32 [1.49] vs 12.90 [1.46] years; *P* = .03), and less education (eg, 17 participants [10.83%] vs 0 participants with no high school diploma; *P* = .004). Of 146 participants with dyslipidemia, 137 (93.8%) had low adherence and only 9 participants (6.2%) had high adherence. Of 103 participants with low adherence to BP-lowering medications and using oral hypoglycemic agents, 83 (80.58%) had low adherence to oral hypoglycemic agents. Beliefs that medications are necessary were higher for participants with high adherence to BP-lowering medications than those with low adherence in unadjusted analyses (mean [SD] necessity score, 16.87 [6.78] vs 13.89 [9.15]; *P* = .03). In adjusted multivariable analyses of participants with hypertension or nephropathy, having at least 1 unmet social need (odds ratio [OR], 0.20; 95% CI, 0.05-0.65; *P* = .04) and medication concerns (OR, 0.63; 95% CI, 0.40-0.96; *P* = .01) were associated with worse medication adherence 1 year follow-up. Diabetes distress, self-efficacy, depressive and anxiety symptoms, and self-management support were not associated with 1-year medication adherence.

**Conclusions and Relevance:**

These findings suggest that adherence to BP- and lipid-lowering medications was very poor in this cohort. To improve medication adherence and prevent early vascular events, approaches that identify and address medication concerns and unmet social needs are needed.

## Key Points

**Question:**

What are adherence rates to blood pressure– and lipid-lowering pills in young adults with youth-onset type 2 diabetes and hypertension, nephropathy, or dyslipidemia and what factors associated with low adherence?

**Findings:**

In this cohort study among 243 participants with youth-onset type 2 diabetes and hypertension, nephropathy, or dyslipidemia, 80.1% of participants with hypertension or nephropathy and 93.8% of participants with dyslipidemia had low adherence (used <80% of pills). Factors associated with low adherence at 1 year follow-up included having concerns about diabetes medications or at least 1 unmet social need.

**Meaning:**

These findings suggest that interventions that target medication beliefs and unmet social needs are needed to improve medication adherence among young adults with youth-onset diabetes to prevent early diabetes-related complications.

## Teaser

This cohort study assesses adherence and factors associated with adherence to antihypertensive and lipid-lowering medications in young adults with youth-onset type 2 diabetes and diagnoses of hypertension, nephropathy, or dyslipidemia

## Introduction

Youth-onset type 2 diabetes is increasing in prevalence and is associated with accelerated development of microvascular and macrovascular complications.^1,2,3,4,5,6^ Treatment of hypertension and nephropathy with angiotensin converting enzyme inhibitors (ACEIs) and angiotensin receptor blockers (ARBs) and treatment of hyperlipidemia with statins are known to prevent or slow progression of kidney disease and reduce cardiovascular events.^7,8,9^ To reduce early morbidity in young adults with diabetes, these medications need to be used.

Poor adherence to antihypertensive and lipid-lowering medications in adults with diabetes is common, especially with younger age^10,11^ and is associated with increased cardiovascular events and mortality.^9,12,13,14^ Most of the medication adherence literature in diabetes focuses on middle-aged and older adults. To our knowledge, adherence to medications used to treat hypertension, nephropathy, and dyslipidemia in young adults with youth-onset diabetes has not been previously reported.

The Treatment Options for Type 2 Diabetes in Adolescents and Youth (TODAY) study, conducted from 2004 to 2011 among individuals ages 10 to 17 years at enrollment, was a randomized clinical trial of 3 interventions (metformin, metformin plus rosiglitazone, and metformin plus intensive lifestyle intervention) in participants with recently diagnosed youth-onset diabetes, with glycemic control as the primary outcome.^1,15^ During the trial, hypertension, nephropathy, and dyslipidemia were diagnosed and treated with ACEIs and statins. These medications were also provided during the early follow-up period (2011-2014). The study then became observational (2014-2020; known as the *TODAY2 study*), during which time participants received diabetes care in their communities. During this observational phase, TODAY2 participants underwent annual study assessments for diabetes-related outcomes.

iCount, an ancillary TODAY2 study, focused on assessing and understanding medication adherence during the last years of the TODAY2 study (2017-2019), when participants were young adults receiving care in their communities. We previously reported poor adherence to oral hypoglycemic agents (OHAs) and insulin in this cohort.^16,17^ At the end of TODAY2, the mean (SD) age was 26 (2.8) years and 67.5% of participants had hypertension, 51.6% of participants had dyslipidemia, and 54.8% of participants had diabetic kidney disease.^1^ Given the high prevalence of these conditions, it is important to understand adherence to antihypertensive and lipid-lowering medications, since these treatments can reduce early onset cardiovascular events and help forestall the progression to end-stage kidney disease. In this study, we report on adherence to medications to lower blood pressure (BP; ie, ACEIs and ARBs for hypertension and nephropathy) and lipids and describe the factors associated with adherence to BP-lowering medications in this young cohort at high risk of morbidity.

## Methods

This cohort study was approved by institutional review boards at the 15 participating centers. All participants provided written informed consent. This study is reported following the Strengthening the Reporting of Observational Studies in Epidemiology (STROBE) reporting guideline.

### Study Sample

The TODAY and TODAY2 study designs have been previously reported.^1,15^ TODAY enrolled 699 youth ages 10 to 17 years with new-onset diabetes who were obese (ie, body mass index, calculated as weight in kilograms divided by height in meters squared, in the ≥85th percentile) and had negative glutamic acid decarboxylase-65 and tyrosine phosphatase autoantibodies. At the conclusion of TODAY, all participants were eligible for the TODAY 2 study, and 572 participants enrolled. iCount assessed medication adherence during the last years of the TODAY2 study (2017-2019). TODAY2 participants ages 19 to 31 years were eligible to enroll. Hypertension and dyslipidemia were not requirements for study entry. There was independent data collection, including telephone unannounced pill-counts, and access to relevant TODAY2 data.

Participants were defined as having hypertension if hypertension was diagnosed during TODAY or TODAY2 (ie, systolic BP ≥130 mm Hg; diastolic BP ≥80 mm Hg; BP in the ≥95th percentile for age, gender, and height at 2 consecutive annual visits; or a single elevated BP and previously prescribed antihypertensive therapy) and were using antihypertension medications at the iCount baseline visit or were previously diagnosed with hypertension during TODAY or TODAY2, were not taking antihypertension medications, and their BP was elevated at the baseline iCount visit. They were defined as having nephropathy if they had a previous diagnosis of microalbuminuria or macroalbuminuria (urine albumin ≥30 mg/G creatinine on 2 consecutive annual visits or at 1 visit and previously prescribed an ACEI) and elevated urine albumin (≥30 mg/G creatinine) or were using an ACEI or ARB at the baseline iCount visit. Participants were defined as having dyslipidemia if they had a diagnosis of dyslipidemia (triglyceride ≥150 mg/dL or low-density lipoprotein cholesterol ≥130 mg/dL [to convert to millimoles per liter, multiply by 0.0113] at 2 consecutive annual visits, or at 1 visit followed by treatment with a lipid-lowering medication) and were using a lipid-lowering drug or were not using a lipid-lowering drug but had lipid panel results outside of reference ranges at the baseline iCount visit. Race and ethnicity were assessed via self-report and categorized as Hispanic, Non-Hispanic Black, Non-Hispanic White, and other (including American Indian or Alaska Native and Asian). Race and ethnicity were assessed as part of standard reporting for all National Institutes of Health–funded research.

### Assessments

Standardized BP measurements and laboratory tests, including fasting lipid panels and urine albumin to creatinine ratio, were performed in a central laboratory (Northwest Lipid Research Laboratories, University of Washington, Seattle, Washington) as described elsewhere.^15^ After providing informed consent for the iCount study, participants were instructed in the procedures for performing remote unannounced pill counts.

### Unannounced Telephone Pill Counts

Trained staff called participants at unscheduled times (once per month for 3 consecutive months beginning 1-2 weeks after iCount enrollment [baseline] and once per month 10, 11, and 12 months later [follow-up]). Participants provided pharmacy label information on all possessed pill dispensers, including number of pills prescribed, dose in milligrams, number of refills, and refill date. Participants counted pills in their possession aloud twice. If the 2 counts differed, staff reviewed procedures to ensure correct counting. Pill counting requires ability to count, not mental calculations.

Unannounced pill counts have been used extensively in research with persons with HIV, including young adults.^18^ Adherence scores using this method were significantly associated with HIV viral load, establishing criterion validity.^19^ Unannounced phone pill counts are valid and reliable compared with in-person and scheduled home pill counts.^20,21,22^ We chose this innovative approach because of its rigor and to avoid bias and validity concerns noted for self-report questionnaires.^23^

### Main Measures

The adherence score (percentage adherence) was calculated by comparing the number of pills counted at 1 time point with the number counted at an earlier time point, considering the number of pills prescribed and dispensed. Participants were contacted for 3 baseline pill counts (months 1, 2, 3). If they completed all 3, we calculated the mean from 2 adherence scores (1 vs 2 and 2 vs 3). We did the same for months 10, 11, and 12, yielding a follow-up adherence score. A score of less than 80% was considered low adherence and 80% or greater, high adherence.

Psychosocial measures were completed at the iCount enrollment visit (baseline) and subsequent 1-year follow-up visit, each with excellent reliability and validity. Perceived seriousness of diabetes, its psychosocial impact, and attitudes toward patient autonomy were measured using 3 subscales in the Diabetes Attitudes Scale (subscale range, 1-5; higher score on each subscale indicates greater belief that diabetes is a serious disease, that diabetes has had a greater impact on quality of life, and that persons with diabetes have a right to decide how hard they will work to control their blood glucose, respectively).^24^ We used the Beliefs About Medicines Questionnaire,^25^ which consists of 2 scales to measure beliefs in the necessity of and concerns about diabetes medicines (range, 5-25; a higher score on each subscale indicates more belief that diabetes medicines are necessary and respondent has more concerns about them, respectively) and 2 scales to measure beliefs that, in general, medicines harm or are overused (range, 4-20; higher score indicates more belief that medicines, in general, are harmful or overused). The Self-Efficacy to Manage Diabetes Scale assesses confidence in one’s ability to perform self-care behaviors (range, 8-80; higher score indicates greater feelings of diabetes self-efficacy).^26^ We used the 5-item Problem Areas in Diabetes Scale to measure diabetes-related emotional distress (range, 0-20; a score ≥8 indicates high diabetes distress).^27^ The Patient Health Questionnaire–8-item (PHQ-8)^28,29^ measures presence and severity of depressive symptoms over the past 2 weeks (range, 0-20; score ≥10 indicates moderate to severe depressive symptoms).^28,29^ The Generalized Anxiety Disorders Questionnaire–7-item (GAD-7).^30,31,32^ Scores range from 0 to 21, with a score of 10 or greater indicating moderate to severe anxiety symptoms.^30,31,32^ To assess resources that support self-care, we used the Chronic Illness Resources Survey^33,34^; respondents indicate the extent to which they have used each resource over the past 6 months (range, 1-5; higher score indicates greater use of support for diabetes self-management). Finally, the Material Needs Insecurities Survey^35^ was used to assess housing, food, and medication insecurities. Respondents indicate whether needs were met or not met due to cost over the previous 12 months. We added a fourth insecurity, lack of health care coverage, as a binary variable.

### Statistical Analysis

In descriptive analyses, categorical data are presented as number and percentage and continuous data as means with SDs. Characteristics of participants with low vs high adherence were compared using Fisher exact test (categorical) and *t* tests (continuous). *P* values were 2-sided, and *P* = .05 was considered statistically significant. Multiple logistic regression analyses were conducted to investigate associations between psychosocial characteristics at baseline and 1 year later adjusting for age, duration of diabetes, education, HbA_1c_, and baseline adherence. Covariates were selected based on bivariate association results. Multiple regression results are presented using odds ratios (ORs), 95% CIs, and *P* values. Health care coverage insecurity, PHQ-8 scores, and GAD-7 scores were removed from the logistic regression analysis due to low sample size. The significance level was not adjusted for multiple testing. All analyses are exploratory. Analyses were performed using R statistical software version 4.0.3 (R Project for Statistical Computing) and SAS statistical software version 9.4 (SAS Institute). Data were analyzed from September 2022 to September 2023.

## Results

**Figure 1.**
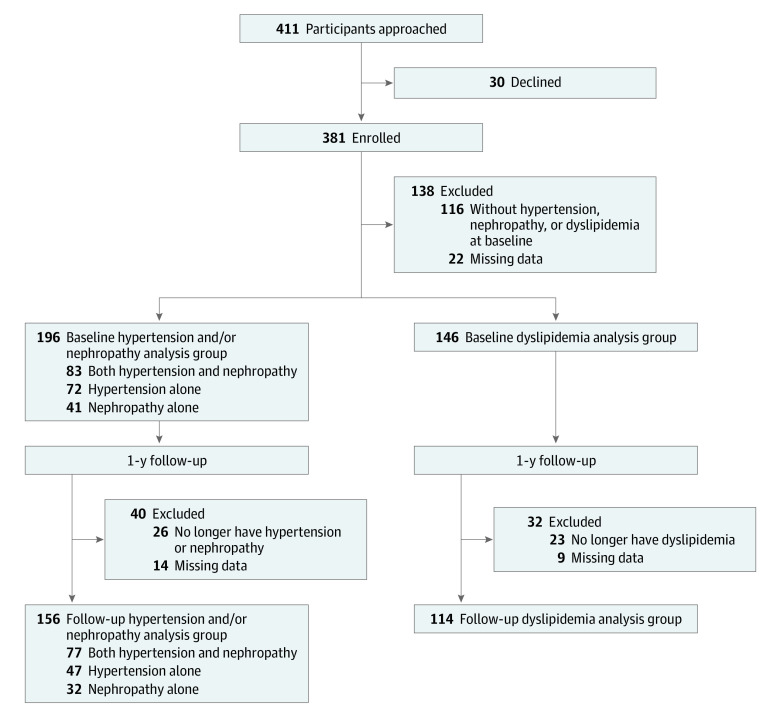
Participant Enrollment The iCount participants and all the participants originally enrolled in the TODAY randomized clinical trial did not differ at baseline by age, gender, race, ethnicity, body mass index, diabetes duration, or glycated hemoglobin A_1c_.

Of 381 iCount participants, 243 participants (mean [SD] age, 26.12 [2.51] years; 159 [65.43%] women) with hypertension, nephropathy, or dyslipidemia were included in analysis (Figure 1). Of these, 196 participants (123 [62.76%] women; mean [SD] age, 26.24 [2.45] years) had hypertension or nephropathy and 146 participants had dyslipidemia (mean [SD] age, 26.03 [2.50]; 98 [67.12%] women) and were included in analyses; 99 participants with dyslipidemia also had hypertension or nephropathy.

Baseline characteristics of participants with hypertension or nephropathy are shown in Table 1. These participants were racially and ethnically diverse, with 67 Hispanic participants (34.18%), 76 non-Hispanic Black participants (38.78%), 38 non-Hispanic White participants (19.39%), and 15 participants (7.65%) with other race or ethnicity. Both nephropathy and hypertension are treated with ACEIs or ARBs; since many participants had both conditions (83 participants [43.35%]) and the number of participants with nephropathy alone (41 participants [20.92%]) was relatively small, participants with hypertension and/or nephropathy are grouped together for these analyses.

**Table 1.** Baseline Characteristics of Participants With Hypertension or Nephropathy

Characteristic	Participants with hypertension or nephropathy, No. (%)			*P* value^b^
	Total (N = 196)	High adherence (n = 39)^a^	Low- adherence (n = 157)^a^	
Age, mean (SD), y	26.24 (2.45)	27.26 (2.41)	25.99 (2.41)	.005
Gender				
Women	123 (62.76)	23 (58.97)	100 (63.69)	.58
Men	73 (37.24)	16 (41.03)	57 (36.31)	
Race and ethnicity				
Hispanic	67 (34.18)	11 (28.21)	56 (35.67)	.45
Non-Hispanic Black	76 (38.78)	15 (38.46)	61 (38.85)	
Non-Hispanic White	38 (19.39)	11 (28.21)	27 (17.20)	
Other^c^	15 (7.65)	2 (5.13)	13 (8.28)	
Education				
<High school diploma	17 (8.67)	0	17 (10.83)	.004
High school or trade school	143 (72.96)	26 (66.67)	117 (74.52)	
≥Associate’s degree	36 (18.37)	13 (33.33)	23 (14.65)	
Annual income, $				
<34 999	149 (83.71)	30 (81.08)	119 (84.40)	.62
≥35 000	29 (16.29)	7 (18.92)	22 (15.60)	
Employment status				
Employed or student	142 (72.45)	30 (76.92)	112 (71.34)	.55
Unemployed or disabled	54 (27.55)	9 (23.08)	45 (28.66)	
BMI	37.27 (8.85)	38.67 (9.47)	36.91 (8.68)	.30
Diabetes duration, y	12.43 (1.49)	12.90 (1.46)	12.32 (1.49)	.03
HbA_1c_, mean (SD), %	10.03 (2.67)	8.85 (2.39)	10.33 (2.66)	.001
Adherence to OHAs^d^				
High	39 (29.32)	19 (63.33)	20 (19.42)	<.001
Low	94 (70.68)	11 (36.67)	83 (80.58)	
Comorbidities or complications	3.07 (1.15)	3.13 (1.10)	3.06 (1.16)	.72
Diabetes in nuclear family	113 (59.16)	25 (64.10)	88 (57.89)	.59
Health care coverage				
No	32 (16.33)	4 (10.26)	28 (17.83)	.34
Yes	164 (83.67)	35 (89.74)	129 (82.17)	

Abbreviations: BMI, body mass index (calculated as weight in kilograms divided by height in meters squared); HbA_1c_, glycated hemoglobin A_1c_; OHA, oral hypoglycemic agent.

^a^ High adherence indicates a participant used at least 80% of medication. Low adherence indicates a participant used less than 80% of medication.

^b^ *P* values were calculated using *t* test (continuous variables) or Fisher exact test (categorical variables).

^c^ Includes American Indian and non-Hispanic Asian individuals.

^d^ Among 133 participants in the hypertension or nephropathy group.

Of 93 participants prescribed pills for hypertension or nephropathy, 85 (91.40%) were using an ACEI or ARB; others were using a calcium channel blocker (1 participant [1.07%]), β-blocker (4 participants [4.30%]), diuretic and β-blocker (1 participant [1.07%]), calcium channel blocker with or without β-blocker and other (2 participants [2.15%]). There were 74 participants (79.57%) using only 1 medication, 16 participants (17.20%) using 2 medications, and 3 participants (3.23%) using 3 medications. Of 32 participants with dyslipidemia using medications, 1 was using gemfibrozil and the rest were using a statin. Adherence was assessed at baseline and 1 year later. There were no significant changes in the percentages of participants in low adherence and high adherence groups from baseline to 1 year.

For participants with hypertension or nephropathy, 157 (80.10%) had low adherence and 39 participants (19.90%) had high adherence. Of participants with low adherence, 106 (67.52%) were not using any BP-lowering medication. Among participants with hypertension alone, nephropathy alone, or both conditions, the percentage of participants with low adherence was greatest in the nephropathy alone group (38 of 41 participants [92.68%]) and the percentage of participants with low adherence was lowest in the group with both hypertension and nephropathy (59 of 83 participants [71.08%]). Of 72 participants with hypertension alone, 60 (83.33%) had low adherence (*P* = .12). For 146 participants with dyslipidemia, 137 (93.84%) had low adherence, of whom 115 participants (83.9%) were not using any lipid-lowering medication; only 9 participants (6.16%) had high adherence.

**Figure 2.**
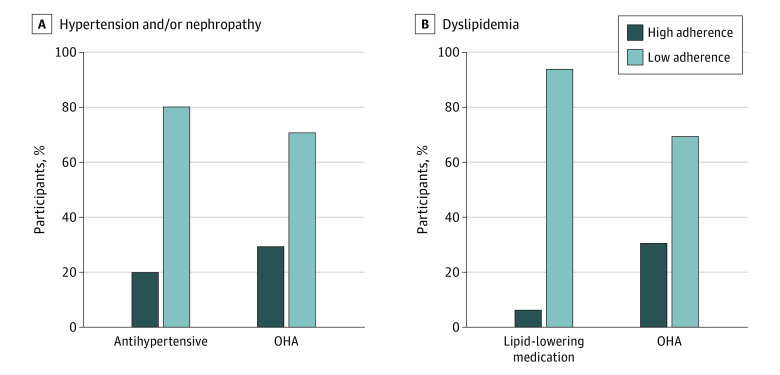
Oral Hypoglycemic Agent (OHA), Blood Pressure–Lowering, and Lipid-Lowering Medication Adherence in Participants With Hypertension, Nephropathy, or Dyslipidemia Low adherence was defined as having used less than 80% of prescribed medication; high adherence, 80% or more of prescribed medication.

We examined adherence to OHAs in participants with hypertension or nephropathy, finding 94 participants (70.68%) had low adherence, similar to our previous iCount reports that included participants with and without comorbidities.^16,36^ As shown in Figure 2, there was better adherence to OHAs than to BP- or lipid-lowering medications. Participants with low adherence to OHAs were significantly more likely to also have low adherence to BP- and lipid-lowering medications (Table 1).

The characteristics of participants with low adherence to BP-lowering medications were compared with participants with high adherence (Table 1). At baseline, participants with low adherence were younger (mean [SD] age, 25.99 [2.41] vs 27.26 [2.41] years; *P* = .005), had higher HbA_1c_ (mean [SD], 10.33% [2.66 percentage points] vs 8.85% [2.39 percentage points]; *P* = .001), and were more likely to have low adherence to OHAs. Participants with low adherence, compared with those with high adherence, also had lower educational attainment (eg, 17 participants [10.83%] vs 0 participants with no high school diploma; *P* = .004) and shorter diabetes duration (mean [SD], 12.32 [1.49] vs 12.90 [1.46] years; *P* = .03). For participants without health care coverage, only 4 had high adherence to ACEIs or ARBs, but since only 32 participants (16.33%) lacked health care coverage, comparisons between those with and without coverage were limited by the low total number of participants with high adherence (Table 1). Given the low number of participants with high adherence to lipid-lowering medications (9 participants), additional analyses were also not possible for this group.

Looking at psychosocial factors, in unadjusted analyses, mean scores for beliefs that medications are necessary were higher in participants with high adherence to BP-lowering medications than in those with low adherence (mean [SD] necessity score, 16.87 [6.78] vs 13.89 [9.15]; *P* = .03) (Table 2). There were no differences between adherence groups for diabetes distress, self-efficacy, depression, anxiety, or self-management support (Table 2).

**Table 2.** Baseline Participant Psychosocial Characteristics

Factor	Score, mean (SD)			*P* value
	Total (N = 196)	High adherence (n = 39)^a^	Low adherence (n = 157)^a^	
**Psychological**				
Diabetes attitudes^b^				
Seriousness of diabetes	4.09 (0.49)	4.12 (0.46)	4.09 (0.49)	.66
Psychosocial impact	4.00 (0.57)	4.00 (0.59)	4.00 (0.57)	>.99
Patient autonomy	3.70 (0.51)	3.64 (0.52)	3.72 (0.51)	.40
Beliefs about medicines^c^				
Specific				
Necessity	14.48 (8.80)	16.87 (6.78)	13.89 (9.15)	.03
Concerns	10.33 (6.79)	11.46 (5.00)	10.04 (7.15)	.15
General				
Harm	9.55 (2.91)	8.95 (2.37)	9.70 (3.01)	.10
Overuse	9.84 (2.97)	9.69 (2.68)	9.88 (3.05)	.71
Diabetes self-efficacy^d^	53.86 (16.23)	56.67 (14.40)	53.16 (16.62)	.19
Diabetes distress^e^				
Score	4.87 (4.79)	4.38 (4.37)	4.99 (4.89)	.45
Participants with high diabetes distress, No. (%)	50 (25.51)	7 (17.95)	43 (27.39)	.31
Depression^f^				
Score	2.74 (3.64)	2.81 (2.77)	2.72 (3.82)	.87
Severity, No. (%)				
None to mild	181 (93.78)	37 (100)	144 (92.31)	.13
Moderate to severe	12 (6.22)	0	12 (7.69)	
Anxiety^g^				
Score	2.15 (3.55)	2.19 (3.15)	2.13 (3.65)	.93
Severity, No. (%)				
None to mild	184 (95.34)	36 (97.30)	148 (94.87)	>.99
Moderate to severe	9 (4.66)	1 (2.70)	8 (5.13)	
**Social**				
Self-management support^h^	2.57 (0.70)	2.63 (0.60)	2.56 (0.73)	.53
Prevalence of material need insecurity, No. (%)^i^				
Medication	39 (35.78)	9 (31.03)	30 (37.50)	.65
Food	77 (46.39)	15 (48.39)	62 (45.93)	.84
Housing	56 (28.57)	9 (23.08)	47 (29.94)	.44
Health care coverage	32 (16.33)	4 (10.26)	28 (17.83)	.34
≥1 Insecurity^j^	117 (73.58)	25 (75.76)	92 (73.02)	.83
≥2 Insecurities^j^	56 (32.75)	8 (22.86)	48 (35.29)	.23

^a^ High adherence indicates a participant used at least 80% of medication. Low adherence indicates a participant used less than 80% of medication.

^b^ Assessed using the Diabetes Attitudes Scale (subscale range, 1-5; higher score on each subscale indicates greater belief that diabetes is a serious disease, that diabetes has had a greater impact on quality of life, and that persons with diabetes have a right to decide how hard they will work to control their blood glucose, respectively).

^c^ Assessed using the Beliefs About Medicines Questionnaire: 2 scales to measure beliefs in the necessity of and concerns about diabetes medicines (range, 5-25; a higher score on each subscale indicates more belief that diabetes medicines are necessary and respondent has more concerns about them, respectively) and 2 scales to measure beliefs that, in general, medicines harm or are overused (range, 4-20; higher score indicates more belief that medicines are harmful or overused).

^d^ Assessed using the Efficacy to Manage Diabetes Scale (range, 8-80; higher score indicates greater feelings of diabetes self-efficacy).

^e^ Assessed using the Problem Areas in Diabetes Scale (range, 0-20; a score ≥8 indicates high diabetes distress).

^f^ Assessed using the Patient Health Questionnaire–8-item (range, 0-20; score ≥10 indicates moderate to severe depressive symptoms).

^g^ Assessed using the Generalized Anxiety Disorders Questionnaire–7-item (range, 0-21; score ≥10 indicates moderate to severe anxiety symptoms).

^h^ Assessed using the Chronic Illness Resources Survey (range, 1-5; higher score indicates greater use of support for diabetes self-management).

^i^ Assessed using the Material Needs Insecurities Survey. Data are presented for participants who reported the insecurity was present.

^j^ Included participants who had missing medication insecurity data but had reported 1 or more food, housing, or health care coverage insecurities.

Longitudinal analyses were performed for participants with hypertension or nephropathy at baseline and 1 year later (156 participants with hypertension and nephropathy; 26 participants with only hypertension or only nephropathy at baseline only). In multivariable analyses adjusting for age, duration of diabetes, education, HbA_1c_, and baseline BP medication adherence among 156 participants with hypertension or nephropathy at baseline and 1 year later, the baseline characteristics associated with low medication adherence 1 year later were having concerns about medications (OR, 0.63; 95% CI, 0.40-0.96; *P* = .04) and at least 1 unmet social need (OR, 0.20; 95% CI, 0.05-0.65; *P* = .01) (Table 3).

**Table 3.** Adjusted Associations of Psychosocial Factors With Blood Pressure Medication Adherence Among Participants With Hypertension or Nephropathy at 1 Year

Factor	Adjusted odds ratio (95% CI)^a^	*P* value
**Psychological **		
Diabetes attitudes, per 1-point increase^b^		
Seriousness of diabetes	0.73 (0.24-2.21)	.57
Psychosocial impacts	0.96 (0.42-2.29)	.93
Patient autonomy	1.35 (0.53-3.49)	.54
Beliefs about medicines, per 5-point increase^c^		
Specific		
Necessity	0.90 (0.66-1.24)	.51
Concerns	0.63 (0.40-0.96)	.04
General		
Harm	1.56 (0.62-3.93)	.34
Overuse	1.15 (0.47-2.77)	.76
Diabetes self-efficacy, per 5-point increase^d^	1.04 (0.88-1.22)	.65
Diabetes distress^e^		
Per 1-point increase	0.90 (0.79-1.01)	.08
Score ≥8 (vs score <8)	0.64 (0.17-2.11)	.48
**Social **		
Self-management support, per 1-point increase^f^	1.11 (0.54-2.24)	.77
Material need insecurity^g^		
Medication	0.32 (0.07-1.21)	.11
Food	0.42 (0.13-1.25)	.13
Housing	0.68 (0.20-2.05)	.51
≥1 Insecurity	0.20 (0.05-0.65)	.01
≥2 Insecurities	0.53 (0.15-1.67)	.29

^a^ Of 156 participants, 30 (19%) had high adherence (used ≥80% of medication) and 126 (81%) had low-adherence (used <80% of medication). Depressive symptoms, anxiety symptoms, and health care coverage insecurity were removed from the regression analysis due to low sample size. All models were adjusted for age, duration of diabetes, education, hemoglobin A_1c_, and baseline blood pressure medication adherence.

^b^ Assessed using the Diabetes Attitudes Scale (subscale range, 1-5; higher score on each subscale indicates greater belief that diabetes is a serious disease, that diabetes has had a greater impact on quality of life, and that persons with diabetes have a right to decide how hard they will work to control their blood glucose).

^c^ Assessed using the Beliefs About Medicines Questionnaire: 2 scales to measure beliefs in the necessity of and concerns about diabetes medicines (range, 5-25; a higher score on each subscale indicates more belief that diabetes medicines are necessary and respondent has more concerns about them, respectively) and 2 scales to measure beliefs that, in general, medicines harm or are overused (range, 4-20; higher score indicates more belief that medicines are harmful or overused).

^d^ Assessed using the Efficacy to Manage Diabetes Scale (range, 8-80; higher score indicates greater feelings of diabetes self-efficacy).

^e^ Assessed using the Problem Areas in Diabetes Scale (range, 0-20; a score ≥8 indicates high diabetes distress).

^f^ Assessed using the Chronic Illness Resources Survey (range, 1-5; higher score indicates greater use of support for diabetes self-management).

^g^ Assessed using the Material Needs Insecurities Survey. Comparisons are among participants reporting the insecurity vs those not reporting the insecurity.

## Discussion

This cohort study addresses gaps in knowledge concerning medication adherence behaviors of young adults with youth-onset diabetes complicated by hypertension, nephropathy, or dyslipidemia. Our findings document poor adherence to BP- and lipid-lowering medications in the TODAY2 cohort and have potential adverse consequences for the early development and progression of kidney and cardiovascular disease (CVD). At the end of TODAY2, 54% of participants had 2 or more CVD risk factors in addition to diabetes.^37^ Elevated concentrations of cardiac biomarkers, worsening arterial stiffness, abnormal heart rate variability, adverse echocardiographic measures, and increases in inflammatory markers over time were described in the TODAY study, further supporting heightened CVD risk.^38,39,40,41^

Low adherence was more common for BP- and lipid-lowering medications in our cohort of young adults with youth-onset diabetes compared with previous reports of adults with adult-onset diabetes who were mostly middle aged or older.^14,42,43,44^ In US adults aged older than 44 years participating in the Medical Expenditure Panel Survey,^14^ 87% of whom had diabetes, 19.5% of participants did not continually use glucose-lowering medications, 17.1% of participants did not continually use antihypertensives, and 43.2% of participants did not continually use lipid-lowering medications. In another study of adults with diabetes without coronary heart disease,^42^ 85% of whom were aged at least 45 years, 37.6% of participants had high adherence to statins, based on pharmacy claims. For young adults in iCount, only 6% of participants had high adherence to lipid-lowering medications. Many of these participants were given ACEIs and statins during the TODAY study but did not persist in using them as young adults, when the study was no longer providing diabetes care.

We previously reported poor adherence to OHAs in iCount.^16,17^ OHA adherence was similar in this study, which only included iCount participants who also had hypertension, nephropathy, or dyslipidemia. To our knowledge, this is the first study in young adults with youth-onset diabetes that found that adherence to BP- and lipid-lowering mediations was worse than for OHAs.

The only longitudinal factors associated with low adherence to BP-lowering medications, after adjustment, were having concerns about medications and having at least 1 unmet social need. In a study of medication adherence in adults on Medicaid with hypertension (80% of participants aged ≥40 years),^45^ low adherence was also associated with food and housing insecurities. We previously reported that low adherence to OHAs was associated with having housing insecurity, 2 or more unmet social needs, and concerns that medications are harmful.^36^ In a study of adults (mean age, 57 years) prescribed diabetes and hypertension medications,^46^ 31.1% of participants underused BP-lowering medications, and underuse was associated with being younger and having beliefs that medicines are harmful. Participants reported believing that glucose-lowering medications were more necessary and more concerning than BP-lowering medications but with a small effect size.^46^

Clinician-level factors could also contribute to underuse of medications for hypertension, nephropathy, and dyslipidemia. We wondered whether clinicians were selectively underprescribing ACEIs or ARBs to female participants, all of whom were of child-bearing age, for fear of teratogenicity in unplanned pregnancies, but there was no difference in percentage of low adherence comparing female participants with male participants. We did not study clinician behavior and do not know whether there was clinical inertia, interfering beliefs, or statins or ACEIs or ARBs were suggested or prescribed but not used by the patient. We doubt that cost was an important contributing factor, since these medications are inexpensive.

Diabetes distress, self-efficacy, depressive and anxiety symptoms, and self-management support were not associated with 1-year medication adherence. This is despite the fact that high distress was found in 24% of participants at the end of TODAY2, and significant depressive symptoms (increased to 19% by the end of TODAY2) were associated with higher HbA_1c_ and hypertension.^47,48^ During the TODAY trial, when OHAs were being provided and incentives were given for adherence, baseline depression was associated with lower OHA adherence.^49^ We did not find this association in the iCount study for OHAs^17^ or BP-lowering medications, when adherence was measured when these young adults were receiving their diabetes care in their communities. However, while pill counts were completed over 3 months, the PHQ-8 and GAD-7 queries about depressive and anxiety symptoms experienced by the participant within the past 2 weeks. This narrower time frame may have failed to capture an association between these variables.

Unlike most reports of medication use in adults with diabetes that are dependent on self-report or pharmacy data, a strength of iCount was that adherence was assessed with unannounced pill counts. Importantly, participants were part of a well-characterized, diverse cohort of young adults with youth-onset diabetes at high risk for developing serious diabetes complications.

### Limitations

This study has some limitations. Participants had been volunteers in the TODAY clinical trial. Eligibility criteria for the TODAY trial included high adherence (≥80%) to metformin treatment during the 8-week run-in period. During the trial, medications (ie, metformin, insulin, ACEIs, and statins) were provided, and participants received education about the importance of medication use during frequent visits over many years. We speculate that young adults who did not have the benefit of that support and education would have worse medication adherence. The small number of participants with dyslipidemia and high adherence limited comparisons between those with high vs low adherence to lipid-lowering medications, and there was only a small number of participants with significant depression and anxiety symptoms. We also did not have access to number or type of daily medications used or out-of-pocket costs.

## Conclusions

This cohort study found that adherence to BP- and lipid-lowering medications was poor and associated with beliefs about medications and social factors in young adults with youth-onset diabetes. Implementing close and continuous monitoring for young adults with unmet social needs to ensure a consistent medication supply and improve adherence could be considered. Understanding all factors affecting medication-taking, including addressing beliefs, is needed to inform future interventions to improve medication use in this group who are at high risk for CVD.

## Notes

Supplement.
Data Sharing Statement
