# Supplementary material for: Antihypertensive and Lipid-Lowering Medication Adherence in Young Adults With Youth-Onset Type 2 Diabetes
Source: JAMA Netw Open. 2023 Oct 4;6(10):e2336964. doi: 10.1001/jamanetworkopen.2023.36964 (PMC10551772; doi:10.1001/jamanetworkopen.2023.36964)
Supplement: Supplement. — Data Sharing Statement [file jamanetwopen-e2336964-s001.pdf]

Data Sharing Statement

Weinstock. Antihypertensive and Lipid-Lowering Medication Adherence in Young Adults With Youth-Onset Type 2 Diabetes. *JAMA Netw Open*. Published October 04, 2023. doi:10.1001/jamanetworkopen.2023.36964

Data
Data available: Yes
Data types: Deidentified participant data, Data dictionary
How to access data: Request for deidentified data should be sent to the study PI, Paula Trief PhD, at triefp@upstate.edu
When available: With publication

Supporting Documents
Document types: None

Additional Information
Who can access the data: Researchers whose proposed use of the data has been approved.
Types of analyses: Data will be made available for analyses that have pre-specified relevant hypotheses
Mechanisms of data availability: Data will be made available after a proposal has been approved and with a signed data access agreement.
Any additional restrictions: None
